# Seroepidemiological Assessment of *Bordetella pertussis* in Jahrom, Southern Iran: A Cross‐Sectional Study

**DOI:** 10.1002/hcs2.70000

**Published:** 2025-02-17

**Authors:** Rahim Raufi, Fatemeh Zareian‐Jahromi, Saba Zangeneh, Jalil Rajabi, Reza Shahriarirad

**Affiliations:** ^1^ Division of Infectious Disease, Department of Internal Medicine Shiraz University of Medical Sciences Shiraz Iran; ^2^ Schol of Medicine Jahrom University of Medical Sciences Jahrom Iran; ^3^ School of Medicine Fasa University of Medical Sciences Fasa Iran; ^4^ Infectious Diseases Research Center AJA University of Medical Sciences Tehran Iran; ^5^ Thoracic and Vascular Surgery Research Center Shiraz University of Medical Sciences Shiraz Iran; ^6^ School of Medicine Shiraz University of Medical Sciences Shiraz Iran

**Keywords:** *Bordetella pertussis*, Iran, seroepidemiology

## Abstract

**Background:**

*Bordetella pertussis*, the causative agent of whooping cough, is a significant contributor to recurrent persistent cough across all age groups, including vaccinated individuals. This seroepidemiological study aims to address the gap in understanding pertussis incidence by investigating its occurrence in individuals with persistent cough and describing the characteristics of affected patients admitted to clinical centers in Jahrom, Southwest Iran.

**Methods:**

This cross‐sectional study enrolled 110 patients with a cough persisting for at least 2 weeks, admitted to clinical centers in Jahrom, Iran. Blood samples were collected at baseline and on day 21 of follow‐up. Serum samples were analyzed for anti‐pertussis toxin immunoglobulin G (anti‐PT‐IgG) levels using an enzyme‐linked immunosorbent assay. Demographic factors, including age, gender, occupation, area of residence, and family size, were also evaluated.

**Results:**

Among the 110 participants, 77 (70%) were female, and seven patients (6.4%) tested seropositive for anti‐PT‐IgG. No significant associations were observed between pertussis incidence and the analyzed variables, including age, gender, occupation, area of residence (urban vs. rural), and family size (*p* > 0.05). These findings underscore the importance of enhancing vaccination coverage to reduce the prevalence of *B. pertussis* in the community.

**Conclusion:**

This study highlights the occurrence of pertussis in individuals presenting solely with a persistent cough, absent of classic symptoms. The findings emphasize the need for healthcare providers to conduct detailed assessments and utilize rapid diagnostic methods for timely detection. This is particularly crucial in regions with high vaccination rates but limited awareness of pertussis recurrence.

## Graphical abstract

Incidence of pertussis based on anti‐pertussis toxin immunoglobulin G among patients referring to infectious clinical centers in Jahrom, Southern West Iran.

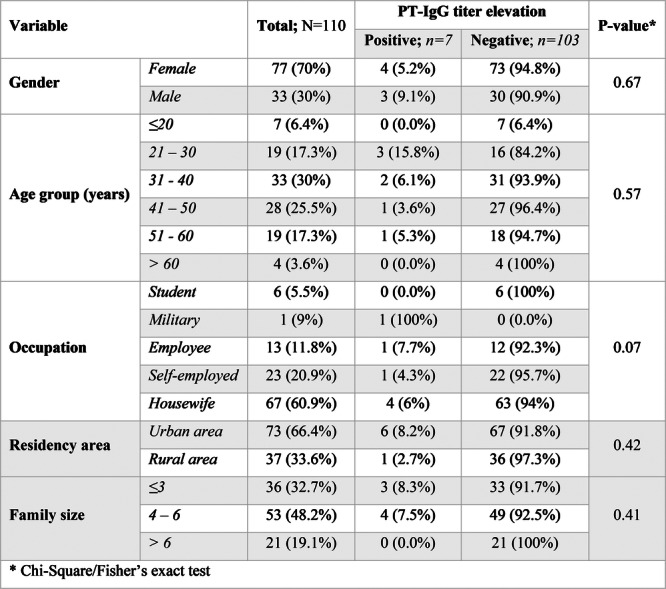

**Abbreviations**
anti‐PT‐IgG: anti‐pertussis toxin immunoglobulin G
DTwP: Diphtheria–Tetanus–Pertussis (whole‐cell vaccine)
ELISA: enzyme‐linked immunosorbent assay
IgG: immunoglobulin G
IU: international units
NITAG: National Immunization Technical Advisory Group
PCR: polymerase chain reaction
PT: pertussis toxin

## 1 Background

Pertussis, commonly known as whooping cough, is a highly contagious respiratory disease caused by the Gram‐negative bacterium *Bordetella pertussis*. It is a leading cause of persistent coughing across all age groups, including vaccinated individuals. The risk of infection increases over time postvaccination [1, 2], primarily due to the limited duration of vaccine‐induced immunity, which lasts up to 12 years. As a result, while vaccinated children generally retain immunity, adults either lose immunity over time or show a weakened immune response [3].

Globally, an estimated 16 million pertussis cases occur annually, with 95% reported in developing countries, resulting in approximately 400,000 deaths [4, 5]. Epidemic outbreaks typically occur every 3–5 years [6, 7]. In recent years, pertussis incidence rates have risen in several countries, including those with high immunization coverage [8, 9]. The serologic test for anti‐pertussis toxin immunoglobulin G (anti‐PT‐IgG) is considered the gold‐standard diagnostic method, as recommended by the Council of State and Territorial Epidemiologists [10].

Numerous studies have documented an increasing trend in pertussis incidence rates among youth and adults [2, 11]. Serological test results indicate that over 10%**–**20% of adults with persistent cough lasting 2–3 weeks are likely to be infected with pertussis [12, 13]. In a study across 12 European countries, cases in infants under 1 year of age were identified between January 1, 2006, and December 31, 2013. Among 3074 infants with a cough lasting more than 2 weeks and specific clinical symptoms, 3% were diagnosed with pertussis. They concluded that adults could be the primary sources of disease transmission, often overlooked in early diagnoses by physicians [14, 15]. Additionally, a separate investigation by Karagul et al. in Turkey, conducted from October 1, 2010, to May 30, 2011, reported 3 (1.4%) and 15 (7%) positive cases based on culture and polymerase chain reaction (PCR) methods, respectively, among 240 patients aged 10–39 years with persistent cough symptoms lasting at least 2 weeks [16].

The study underscored the importance of thorough investigations into *B. pertussis* in the differential diagnosis of lingering cough in adults, emphasizing that prompt diagnosis and treatment could curb transmission [16]. In 2012, there were reports of an increase in pertussis cases in several countries, which may reflect the situation in Iran [8]. Studies that year were mainly focused on the northern regions, where the number of reported cases of pertussis among the Iranian population was increasing [17]. A hospital study in Tehran showed that among 42 adults with chronic cough suspected of having pertussis, 15% tested positive using PCR [18]. In addition, a study of 288 pregnant women admitted to Iranian hospitals between 2011 and 2012 showed that 35% had evidence of pertussis infection. A study from Fars province has reported a lower prevalence compared with other regions [19, 20]. Our research adds to the 2012 data by looking at the southern regions of Iran and provides a clearer picture of whooping cough prevalence and spread.

In Iran, the vaccination against pertussis plays a crucial role in the National Immunization Program, which is managed by the National Immunization Technical Advisory Group that was founded in 1982. This program involves administration of the Diphtheria–Tetanus–Pertussis vaccine at 2, 4, 6, and 18 months, along with a booster shot at the age of six [21]. While this vaccination schedule has greatly reduced pertussis cases in children, the immunity that it provides diminishes over time, making both adolescents and adults vulnerable to infection [22]. This is particularly concerning because adults can serve as reservoirs for the disease [23], frequently transmitting it to unvaccinated or partially vaccinated infants, who are at a higher risk of experiencing severe complications [20]. To resolve this situation, booster vaccinations are administered to specific high‐risk groups, including military personnel and women of childbearing age, to help limit disease transmission. However, the ongoing incidence of pertussis in older demographics highlights the necessity for enhanced strategies for adult booster vaccinations and ongoing surveillance efforts [22].

Despite its global significance, limited attention has been paid to this subject in the literature, both internationally and in Iran. This study is vital due to the long‐standing impact of pertussis on human health, as it is a highly contagious disease that poses a serious risk of mortality, especially among unvaccinated infants. Although vaccination initiatives in the past have led to a significant decrease in incidence rates, there has been a recent increase in cases, even in areas with high vaccination coverage [24]. Knowing the incidence rate of the disease is important for creating public health policies that protect at‐risk groups. Therefore, our research aims to fill this gap by investigating the pertussis incidence rate in patients admitted to clinical centers in Jahrom, Southern West Iran.

## 2 Methods

### 2.1 Study Design and Population

This descriptive cross‐sectional study included a total of 110 patients who visited clinical centers for infectious diseases affiliated with Jahrom University of Medical Sciences hospitals from January to March 2012. Eligibility criteria required participants to have a persistent cough for more than 2 weeks, based on the onset of symptoms, ensuring that they were at an appropriate stage for detecting serological markers, such as anti‐PT‐IgG. Blood samples were collected after this duration to allow sufficient time for the immune response to develop and improve the accuracy of serological testing. Additionally, participants were required to have no history of vaccinations within the last 5 years to minimize potential interference from vaccine‐induced antibody responses in the serological measurements. The age range of participants was set between 12 and 72 years to ensure a broad yet relevant demographic representation. Individuals with conditions such as allergies, sinusitis, gastroesophageal reflux disease, chronic obstructive pulmonary disease, or asthma, as well as those taking medications, particularly antibiotics, were excluded to reduce confounding factors that could affect the study's serological and clinical data accuracy.

### 2.2 Data Collection and Measurements

Eligible patients provided written consent before the recording of demographic data, including gender, age, occupation, area of residence, and family size. Venous blood samples (2cc) were collected from each patient twice: once at baseline and again on the 21st day of follow‐up. Serum samples were stored at **−**20°C for subsequent serological testing. The concentration of anti‐PT‐IgG in the patients' serum was measured using an enzyme‐linked immunosorbent assay kit from IBL International GmbH Corporate (Cat. No. IB79208). The specified ranges for anti‐PT‐IgG levels were as follows: values above 100 IU/mL indicated recent infection or vaccination; levels below 40 IU/mL suggested the absence of infection; and values between 40 and 100 IU/mL necessitated further testing.

### 2.3 Statistical Analysis

Data were analyzed using IBM SPSS Statistics software (version 26) using descriptive and analytical tests, including the Chi‐square test and Fisher's exact test. Significance levels for all tests were set at *p* < 0.05.

### 2.4 Ethical Considerations

Before participation, patients were informed about the research process, and written informed consent was obtained. The study adhered to the principles of the Helsinki Declaration, ensuring patient privacy at all stages. The study incurred no additional costs for patients. Ethical approval was granted by the Ethics Committee of Jahrom University of Medical Sciences (ethics code: IR.JUMS.REC.1390.020).

## 3 Results

A total of 110 qualified patients were enrolled in the study. Among these, seven patients (6.4%) showed elevated anti‐PT‐IgG titers at days 0 and 21. The specific anti‐PT‐IgG titer values for the seven patients were above 100 IU/mL, indicating recent infection or vaccination. Regarding the other 103 patients, their anti‐PT‐IgG levels were below 40 IU/mL at both days 0 and 21, suggesting the absence of infection. No detectable anti‐PT‐IgG was found in these patients within the specified range. Values between 40 and 100 IU/mL would have required further testing, but none were observed in this cohort.

The gender distribution of the participants showed a higher representation of females. However, despite the higher number of females enrolled, the proportion of males showing elevated PT‐IgG was slightly higher. Of the 33 male patients, 3 (9.1%) had elevated PT‐IgG titers, compared with 4 out of 77 female patients (5.2%). Table 1 demonstrates the incidence of pertussis based on the demographic features in our study.

**Table 1:** Incidence of pertussis based on anti‐pertussis toxin immunoglobulin G (anti‐PT‐IgG) among patients referring to infectious clinical centers in Jahrom, Southern West Iran.

Variable	PT‐IgG titer elevation			*p* value*
	Total; *N* = 110 (%)	Positive; *n* = 7 (%)	Negative; *n* = 103 (%)	
Gender				
Female	77 (70)	4 (5.2)	73 (94.8)	0.67
Male	33 (30)	3 (9.1)	30 (90.9)	
Age group (years)				
≤ 20	7 (6.4)	0 (0.0)	7 (6.4)	0.57
21–30	19 (17.3)	3 (15.8)	16 (84.2)	
31–40	33 (30)	2 (6.1)	31 (93.9)	
41–50	28 (25.5)	1 (3.6)	27 (96.4)	
51–60	19 (17.3)	1 (5.3)	18 (94.7)	
> 60	4 (3.6)	0 (0.0)	4 (100)	
Occupation				
Student	6 (5.5)	0 (0.0)	6 (100)	0.07
Military	1 (9)	1 (100)	0 (0.0)	
Employee	13 (11.8)	1 (7.7)	12 (92.3)	
Self‐employed	23 (20.9)	1 (4.3)	22 (95.7)	
Housewife	67 (60.9)	4 (6)	63 (94)	
Area of residence				
Urban area	73 (66.4)	6 (8.2)	67 (91.8)	0.42
Rural area	37 (33.6)	1 (2.7)	36 (97.3)	
Family size				
≤ 3	36 (32.7)	3 (8.3)	33 (91.7)	0.41
4–6	53 (48.2)	4 (7.5)	49 (92.5)	
> 6	21 (19.1)	0 (0.0)	21 (100)	

The data showed no instances of elevated PT‐IgG among individuals younger than 20 years or older than 60 years of age, suggesting that pertussis primarily affected adults in their middle years in this cohort. The most affected age group was 21**–**30 years, with 3 out of 19 patients (15.8%) demonstrating elevated titers. This was followed by the 31**–**40‐year‐old group, where 2 out of 33 patients (6.1%) had elevated PT‐IgG levels. One patient in the 51**–**60‐year‐old age group (5.3%) tested positive for elevated PT‐IgG. These findings may point to a higher susceptibility or undiagnosed exposure in younger adults, although further analysis is needed.

Regarding occupation, the highest incidence of pertussis infection was among housewives, with four cases (57.14%), followed by self‐employed individuals, accounting for one case (4.3%). Military personnel and employees had slightly higher incidences, with one case each (9% and 7.7%, respectively). None of the students in the study tested positive for pertussis infection.

A higher proportion of pertussis cases was observed among urban residents compared with those living in rural areas. Of the 73 patients residing in urban areas, 6 (8.2%) demonstrated elevated PT‐IgG titers. In contrast, only 1 out of 37 rural residents (2.7%) had elevated PT‐IgG levels. This difference could reflect varying access to healthcare facilities, differential exposure to infection, or underreporting in rural regions. Urban centers may have higher population density, leading to more frequent person‐to‐person transmission of the *B. pertussis* bacterium.

Family size also appeared to play a role, with three cases (8.3%) occurring in families with three or fewer members and four cases (7.5%) occurring in families with 4–6 members. However, there were no statistically significant correlations between the occurrence of pertussis and variables, such as age, occupation, or family size.

## 4 Discussion

In this study, elevated serum IgG titers were observed in 6.4% of patients experiencing cough lasting more than 2 weeks. These cases were observed among adult patients between 20 and 60 years of age. Numerous studies conducted in developed countries have consistently identified pertussis as a common cause of frequent and severe coughing, with an incidence rate ranging from 2.9% to 32% in adults. Unfortunately, physicians often remain unaware of this disease, which leads to its misdiagnosis and transmission [16, 25]. A study by Ghotaslou and Asl revealed an overall *B. pertussis* positivity of 36% in serum samples from Asians between 2000 and 2015, with Iran recording a higher incidence at 38.4% [26]. However, a similar study at the Pasteur Institute in Iran among suspected patients also yielded a 6.6% positivity rate similar to the present study [27]. Notably, regional variations in incidence rates may be influenced by differences in disease epidemiology, sample characteristics, study timing, and age groups. Also, various cut‐off points and different assays may contribute to variations in results and limit comparability [28, 29]. Moreover, the presence of anti‐PT‐IgG seropositivity may result from a past pertussis infection or vaccination [3]. Despite these considerations, the current study found that 93.6% of the tested adults were seronegative, indicating their susceptibility to pertussis infection. Therefore, it cannot be underestimated that pertussis is an important public health concern, warranting continued attention and preventive measures to mitigate its impact on susceptible populations.

In examining the seasonal patterns of *B. pertussis* in Iran compared with other countries, significant differences and similarities are observed. In Iran, confirmed cases of *B. pertussis* primarily affect infants under 6 months of age, especially during specific seasons, with a noticeable increase among unvaccinated children [27, 30]. This finding is consistent with results from other regions, such as Spain, where low levels of maternal antibodies increase the vulnerability of newborns, leading to higher incidence rates during peak seasons [31].

Our study, conducted between January and March 2012, revealed a pattern similar to the longitudinal study carried out in Iran between 2011 and 2013, which identified winter as the season with the highest incidence of clinical *B. pertussis* cases [20]. This consistent winter peak underscores the significant impact of seasonal factors on pertussis transmission in Iran, especially in the cold months when conditions may facilitate the spread of respiratory infections. Understanding this pattern is essential for timely preventive measures, including vaccination and public health attention, to reduce pertussis transmission during the high‐risk period of winter. Conversely, this seasonal variation may differ from countries like the United States, where pertussis outbreaks typically peak in the summer months [32], likely due to differences in vaccination strategies and population demographics.

Pertussis, as mentioned, is considered an important risk factor for children under 6 months, and according to studies, extra attention should be paid to children under 2 months, who are particularly vulnerable to severe forms of the disease [33]. In a study conducted at Beijing Ditan Hospital, 41.85% of the 184 hospitalized infants were under 3 months old, and a significant proportion of them had not received the pertussis vaccine, placing them at risk for severe outcomes, such as hyperleukocytosis and pneumonia [34]. Another study in France revealed that 9% of 361 infants developed fulminant pertussis, with a notable association between the disease and unvaccinated mothers as well as PRN‐producing isolates of *B. pertussis* [33]. These findings underscore the importance of understanding the transmission of pertussis and the urgent need for prompt diagnosis and treatment to reduce the burden of the disease on young infants.

Comparable international studies, such as the one carried out by Pimentel et al. in Brazil [25], reported a pertussis incidence rate of 5.21%, with 10 out of 192 suspected cases confirmed. In contrast, Lee et al. in the United States found a higher incidence rate of 13.6% among 686 coughing patients [10], while Torzsa et al. in Hungary observed a 14.8% seropositivity rate among healthy adults [35]. Despite high childhood vaccination rates in these countries, immunity to pertussis gradually wanes over time [36, 37, 38, 39], making many adults vulnerable to infection. Studies have demonstrated a significant decrease in pertussis antibody levels 5 years postvaccination, with some findings indicating a reduction of over 50% in individuals who received their last immunization more than 4 years back [8]. Addressing this issue could involve the implementation of adult pertussis boosters, a strategy already embraced by several European countries [40] and the United States [41]. This policy has not yet been adopted in our country, Iran. Given the lower seroprevalence observed in our study compared with other studies, heightened awareness and prompt diagnosis could effectively manage the burden of the disease for the time being.

Several factors contribute to coughing in vaccinated populations, including inadequate vaccination, changes in vaccine quality, immunosuppression, demographic shifts, bacterial adaptation, and misdiagnosis. Adolescents and young adults with lower levels of protective antibodies against pertussis toxin may experience recurrence. Despite milder manifestations in older individuals, pertussis in adults can be multifaceted [42]. An analysis of the relationship between age and pertussis incidence rate in the present study did not show statistical significance. Most cases were observed in the second age group (21–30 years old, 15.8%), followed by age group 3 (31–40 years old, 6.1%). Similar to our report, Torzsa et al. also reported seropositivity for pertussis among the 18 to 29, and above 60 age group [35]. Despite the rapid decline in immunity following pertussis vaccination, anti‐PT antibodies can endure for around 10 years [3, 43]. As a result, the elevated rate of seropositivity noted in the younger age groups is likely due to childhood vaccination. In contrast, among older individuals, the increased seropositivity is probably associated with pertussis infection. Pertussis vaccination in Iran is administered up to the age of six for each individual [21]. According to our findings, there were no instances of seropositivity in the under‐20 age group, which falls within the vaccination immunity time frame. However, the absence of seropositivity in this age group could be concerning, as it heightens susceptibility to active infection. Cases of seropositivity were observed in the 20–60 age group, and we believe that this may be attributed to active infection due to compromised immunity in these individuals.

Gender differences were investigated, with a higher pertussis incidence rate in males, although not significantly noticeable. Similar patterns of higher male seropositivity and likelihood of recent infection have been observed in studies from Mexico [44], Republic of Korea [45], and the Netherlands [46], while studies from Spain [47], Greece [48], Hungary [35], Denmark [49], China [49, 50], and Gambia [51] reported no significant gender differences in seropositivity or likelihood of infection. However, Ghorbani et al. emphasize that the influence of varying age groups in recruitment may cause discrepancies among genders [27]. Overall, the existence of a true link between pertussis and gender remains unclear, and if such a link does exist, the underlying cause remains unknown.

Although the incidence of pertussis was higher in urban areas compared with rural districts, the difference was not statistically significant. This could be influenced by factors, such as access to healthcare, timely diagnosis, vaccination coverage, population density, and transmission routes. Similarly, other studies in Iran have also reported no significant differences between rural and urban populations [27].

Our study also examined occupation and family size, finding higher pertussis occurrence among employees, military personnel, and families with one to three members. However, these differences were not statistically significant. These findings are consistent with studies carried out by Lytras et al. and Tarlo et al. which suggest that exposure to environmental factors may contribute to symptoms, such as cough or sputum production [52, 53]. The transmission dynamics of pertussis within households remain poorly understood and warrant further investigation. For instance, a study in South Africa reported a household cumulative infection risk of 14%, while a pertussis outbreak in New South Wales, Australia, in 2001 showed a higher secondary household attack rate of 22.3% [54]. Notably, the risk of household transmission increased when the index case–patient initiated antimicrobial treatment more than 7 days after symptom onset [54]. These findings highlight the complex interplay of environmental and sociodemographic factors in pertussis transmission. A deeper understanding of these dynamics is essential for developing targeted preventive strategies and effective public health interventions.

Noteworthy limitations of our study include the following: The sensitivity of PCR decreases over time, making anti‐PT‐IgG a more reliable diagnostic tool for patients with a cough lasting more than 2 weeks [10]. While PCR is effective in detecting infection during the early stages, its accuracy significantly diminishes after 2 weeks as bacterial levels decline, potentially leading to false‐negative results [55]. In contrast, anti‐PT‐IgG levels remain elevated for a longer duration postinfection, allowing for the identification of cases that PCR might miss [10]. This makes anti‐PT‐IgG particularly useful for diagnosing pertussis in patients with prolonged cough, especially among adults and adolescents with atypical symptoms [56]. In our study, the combination of both PCR and anti‐PT‐IgG could have enhanced diagnostic accuracy, providing a more comprehensive assessment. Additionally, challenges in patient recruitment for sample collection on day 21 were mitigated through frequent follow‐ups and the exclusion of participants unwilling to undergo the second sampling. Finally, larger‐scale population studies are needed to explore the relationship between demographic features and seropositivity through multivariate analysis, which would help identify risk factors and support more targeted interventions.

## 5 Conclusion

The primary objective of this seroprevalence study was to investigate the occurrence of pertussis in individuals with prolonged cough but without typical symptoms. Our findings revealed that 6.4% of the adult population with persistent cough tested seropositive for anti‐PT‐IgG antibodies. Pertussis, a highly contagious acute respiratory infection, often presents as a persistent cough in adults, contributing to its underdiagnosis in the early stages. Additionally, the high seronegativity observed in our study highlights a population at risk of pertussis infection. These results underscore the importance of considering pertussis as a differential diagnosis in cases of persistent cough, even in the absence of classic symptoms, and advocate for increased awareness among healthcare professionals to enable timely identification and management of this condition.

## Author Contributions

**Rahim Raufi:** conceptualization (equal), project administration (equal), writing–review and editing (equal). **Fatemeh Zareian‐Jahromi:** data curation (equal), investigation (equal). **Saba Zangeneh:** validation (equal), writing–review and editing (equal). **Jalil Rajabi:** methodology (equal). **Reza Shahriarirad:** formal analysis (equal), project administration (equal), validation (equal), writing–original draft (equal), writing–review and editing (equal). All authors proofread and approved the final version of the manuscript.

## Ethics Statement

The study adhered to the principles of the Helsinki Declaration, ensuring patient privacy at all stages. The study incurred no additional costs for patients. Ethical approval was granted by the Ethics Committee of Jahrom University of Medical Sciences (ethics code: IR.JUMS.REC.1390.020).

## Consent

Written informed consent was obtained from the patients in our study.

## Conflicts of Interest

The authors declare no conflicts of interest.

## Data Availability

The data that support the findings of this study are available from the corresponding author upon reasonable request.
